# No significant differences in postoperative clinical outcomes evolution after fresh osteochondral allograft transplantation of the knee between patients with pathological and non‐pathological scores regarding anxiety, depression, kinesiophobia and catastrophizing factors

**DOI:** 10.1002/jeo2.70210

**Published:** 2025-03-07

**Authors:** Pablo Eduardo Gelber, Eduard Ramírez‐Bermejo, Anna Castellà‐Pujol, Aránzazu Gonzalez‐Osuna, Oscar Fariñas

**Affiliations:** ^1^ Department of Orthopaedic Surgery Hospital de la Santa Creu i Sant Pau Universitat Autonoma de Barcelona Barcelona Spain; ^2^ ReSport Clinic Barcelona Spain; ^3^ Barcelona Tissue Bank, Banc de Sang i Teixits Barcelona Spain

**Keywords:** allografts, anxiety, cartilage, depression, kinesiophobia

## Abstract

**Purpose:**

The aim of this study was to determine the influence of preoperative psychological factors on clinical outcomes of fresh osteochondral allograft (FOCA) transplantation of the knee. The hypothesis was that patients with preoperative pathological scores on psychological factors would show worsen functional outcomes after FOCA transplantation of the knee.

**Methods:**

A prospective data collection study was performed from patients undergoing FOCA transplantation for osteochondral lesions of the knee. All patients were followed up for 30 months. Psychological factors of anxiety, depression, kinesiophobia and catastrophizing were assessed by means of self‐administered Hospital Anxiety and Depression Subscale (HADS), Tampa Scale for Kinesiophobia (TSK) and Pain Catastrophizing Scale (PCS) questionnaires one week prior to surgery. Clinical outcomes were evaluated preoperatively and at 3, 6, 9, 12, 15 and 30 months postoperatively using the Kujala score, the Western Ontario Meniscal Evaluation Tool (WOMET) score, the International Knee Documentation Committee (IKDC) score and the Tegner Activity Scale. Participants were classified as pathological or non‐pathological scores for each psychological parameter in accordance with the cut‐off point proposed by the authors of each questionnaire. The interaction between clinical outcome's evolution and pathological scores was analysed using two‐way ANOVA tests with Greenhouse–Geisser correction to avoid non‐sphericity errors.

**Results:**

Forty‐one cases were included (mean age 37.1 years old, 41% female). In the postoperative clinical outcome's evolution, no differences were observed between preoperative pathological and non‐pathological scores (*p* > 0.05) regarding anxiety, depression, kinesiophobia and catastrophizing factors.

**Conclusions:**

No significant differences were observed in the evolution of postoperative clinical outcomes between patients with pathological and non‐pathological psychological scores.

**Level of Evidence:**

Level III, case series.

**Abbreviations**
AKP: anterior knee pain
BMI: body mass index
FOCA: fresh osteochondral allograft
HADS: Hospital Anxiety and Depression Subscale
IKDC: International Knee Documentation Committee
PCS: Pain Catastrophizing Scale
SD: standard deviations
TKA: total knee arthroplasty
TSK: Tampa Scale for Kinesiophobia
WOMET: Western Ontario Meniscal Evaluation Tool

## INTRODUCTION

Osteochondral lesions of the knee have a considerable impact on daily living activities [19], leading to pain, swelling of the knee and functional impartment. If these lesions are left untreated, they may increase in size and severity, eventually leading to diffuse osteoarthritis [3]. Fresh osteochondral allograft (FOCA) transplantation is a single‐stage procedure without donor‐site morbidity for the treatment of large osteochondral lesions (>2 cm^2^) in young patients [3, 13, 14, 34]. Transplanting a FOCA involves the transfer of a size‐matched mature hyaline cartilage‐bone block into large osteochondral lesions of the knee. Favourable long‐term clinical and functional outcomes have been reported after FOCA transplantation, with survival at 10 years of 82%, 91.7% and 78.1% for femoral condyle lesions [28], femoral trochlear lesions [2] and patellar lesions, respectively [17]. Several factors have been related with less favourable results, as higher body mass index (BMI), bipolar transplantations and larger osteochondral defects (>10 cm^2^) [7, 10, 32].

It has recently been reported that psychological factors may play an important role in recovery in a wide range of surgeries [11, 27, 30]. Depression, anxiety, kinesiophobia and/or catastrophizing have been associated with worse outcomes and postoperative quality of life in the treatment of chronic lumbar pain [22], anterior knee pain [6] and total knee arthroplasty [24]. Unfortunately, the impact of these psychological factors on FOCA transplantations of the knee has not been assessed yet.

The purpose of this study was then to assess the influence of preoperative depression, anxiety, kinesiophobia and catastrophizing on clinical outcome's evolution after FOCA transplantation of the knee. The hypothesis was that patients with pathological preoperative scores on psychological factors would have worse outcome's evolution after FOCA transplantations of the knee.

## MATERIALS AND METHODS

We performed a prospective case series study that included all consecutive patients undergoing cartilage repair with FOCA transplantation for osteochondral knee lesions between October 2017 and October 2020. All the surgical procedures were carried out by a single surgeon at an academic medical centre.

Inclusion criteria for FOCA transplantation were patients younger than 50 years old who underwent FOCA transplantation for symptomatic osteochondral knee lesions after non‐operative treatments had failed for at least 6 months. FOCA transplantation was indicated in patients with large focal full‐thickness chondral and osteochondral defects ( > 2 cm^2^) on the tibial plateau, femoral condyles, trochlea and/or patella. Exclusion criteria were active neoplasia, large degenerative lesions comprising all three compartments, BMI > 30 kg/m^2^, systemic inflammatory diseases and infection.

The study was approved by the ethics committee of our institution on September 2017 (IIBSP‐ALO‐2018‐21). Informed consent was obtained from each patient following the guidelines laid down by our local ethics committee.

### Psychological evaluation

Psychological questionnaires were given to the patients during their clinical visit after the physician had checked the inclusion criteria and the patient consented to participate. All participants completed the questionnaires on their own and alone, without the presence of staff or accompanying persons.

Participants were classified as having normal, borderline, or pathological scores for each psychological parameter in accordance with the cutoff point proposed by the authors of each questionnaire.

All participants completed the following psychological questionnaires:

The Hospital Anxiety and Depression Subscale (HADS) questionnaire [35] is a self‐assessment scale for use in hospital outpatient departments. It consists of 14 questions, subdivided into items of depression (HADD) and anxiety (HADA) (7 questions for each item). Following Zigmond and Snaith [35], total scores over 11 points are considered to indicate anxiety or depression, while scores of 8–10 points are considered as possible cases and scores of 0–7 points as normal. Spanish version of HADS was used, which had been previously validated and showed favourable sensitivity and specificity in a previous validation [20].

Kinesiophobia, defined as fear of injury due to movement, has shown to have a strong association with pain intensity, posterior disability, and quality of life in patients with chronic musculoskeletal pain [29]. Spanish version of Tampa Scale for Kinesiophobia (TSK) TSK‐11‐V Scale was used [16]. Patients rated 11 statements from 1 (strongly disagree) to 4 (strongly agree). A total score of over 21 was considered as kinesiophobia.

The Pain Catastrophizing Scale (PCS) is defined as maladaptive cognition regarding pain, resulting in excessive rumination, feelings of incapacity and disproportionate anxiety towards activities that can cause pain or negative speculation concerning future clinical evolution [26]. Spanish version of the PCS‐Scale was used [12]. Patients rated items on 13 statements from 0 (not at all) to 4 (all the time) based. A total score of over 24 was considered as pain catastrophizing.

### Functional evaluation

Clinical results were collected preoperatively and at 3, 6, 9, 12, 15 and 30 months postoperatively. At each time point, all participants completed several patient‐reported outcome instruments to measure clinical results. The scores used were the International Knee Documentation Committee (IKDC), Kujala, the Western Ontario Meniscal Evaluation Tool (WOMET), and the Tegner Activity Scale [4, 15, 18, 21, 25, 31].

### Secondary outcomes

Sociodemographic questions were recorded at baseline to characterise the study sample and explore age, sex assigned at birth, side of lesion (left, right) and BMI as potential confounding variables.

### Statistical analysis

Statistical analysis was performed using IBM SPSS V26.0 statistical package (IBM Corporation Armonk, NY). Descriptive statistics were used for each of the measures evaluated. Results were calculated with means and standard deviations (SD) for quantitative variables and number of cases and percentages for categorical variables.

Quantitative dependent variables were categorised into three groups for HAD and two groups for PCS and TSK.

The power calculation was done according to IKDC from preoperative to 30 months postoperatively. A 5‐point threshold for clinical relevance was set a priori. This number is in fact lower than multiple reported studies to detect minimal changes. According to the power calculation, to generate a power of 80%, an alpha of 0.05, and a standard deviation of 10 points, this study required 30 patients.

Variables repeated during the trial (functional scales) were analysed using two‐way analysis of variance (ANOVA) tests for repeated measures with Greenhouse–Geisser correction to avoid non‐sphericity errors.

The interaction between clinical outcome's evolution and pathological scores was analysed using two‐way ANOVA tests with Greenhouse–Geisser correction to avoid non‐sphericity errors. The overall level of significance was set at 0.05.

## RESULTS

Forty‐one patients met the inclusion criteria, and all gave informed consent to participate. Their mean age was 37.1 years (range, 18–48) and 41% were female. The mean postoperative follow‐up was 42 months (range, 30–52 months).

Thirty‐four of the 41 patients (82.9%) received unipolar OCA transplants, defined as involving ≥ one non‐apposing articulating surfaces, and 7 (17.1%) received bipolar transplants, defined as involving two opposing articulating surfaces.

Results showed pathological scores in 29.3% of patients on the HAD anxiety subscale, in 12.2% of patients on the HAD depression subscale, in 14.6% of patients on the TSK, and in 87.8% on the PCS (Table 1).

**Table 1:** Results on psychological scores evaluation.

		Pathological score	Borderline score	Normal score
HADA	*n*	12	8	21
	%	29.3	19.5	51.2
HADD	*n*	5	9	27
	%	12.2	22	65.9
TSK	*n*	6	‐	35
	%	14.6	‐	85.4
PCS	*n*	36	‐	5
	%	87.8	‐	12.2

Abbreviations: HADA, Hospital Anxiety and Depression‐Anxiety; HADD, Hospital Anxiety and Depression‐Depression; PCS, Pain Catastrophizing Scale; TSK, Tampa Scale for Kinesiophobia.

Preoperative and postoperative comparisons of clinical scores at 3, 6, 9, 12, 15 and 30 months postoperatively showed a significant continuous improvement in IKDC, Kujala and WOMET Scores (*p* < 0001). The Tegner scale showed a return to pre‐injury scores (*p* < 0.659) (Table 2).

**Table 2:** Clinical outcomes.

	Preop	3 months	6 months	9 months	12 months	15 months	30 months	Greenhouse–Geisser, *p* value
IKDC	31.47 ± 13.57	41.87 ± 13.4	48.63 ± 16.7	51.4 ± 15.5	54.4 ± 16.8	55 ± 20.6	58.9 ± 21.1	<0.001
Kujala	41.7 ± 16.8	51.24 ± 16	60.24 ± 18.3	61.53 ± 16.9	63.8 ± 16.9	63.08 ± 18.9	67.5 ± 20	<0.001
WOMET	53.6 ± 26.1	49.7 ± 20	53.79 ± 19	58.11 ± 20.3	64.03 ± 20.3	61.5 ± 24.4	64.9 ± 26.3	0.007
Tegner	2.3 ± 1.4	2.11 ± 1.2	2.3 ± 1.5	2.79 ± 1.2	2.63 ± 1.2	2.45 ± 1.2	2.55 ± 1.2	0.658

*Note*: The values are given as mean ± SD.

Abbreviations: IKDC, the International Knee Documentation Committee; SD, standard deviation; WOMET, Western Ontario Meniscal Evaluation Tool.

After a minimum follow‐up of 2 years, no differences were observed in clinical outcome's evolution between patients with and patients without preoperative pathological scores (Table 3).

**Table 3:** Interaction between clinical outcome's evolution with psychological outcomes.

Significance (p)	IKDC	Kujala	WOMET	Tegner
HADA	0.578	0.117	0.256	0.281
HADD	0.716	0.817	0.662	0.842
TSK	0.498	0.544	0.061	0.104
PCS	0.713	0.988	0.717	0.816

Abbreviations: HADA, Hospital Anxiety and Depression‐Anxiety; HADD, Hospital Anxiety and Depression‐Depression; IKDC, the International Knee Documentation Committee; PCS, Pain Catastrophizing Scale; TSK, Tampa Scale for Kinesiophobia; WOMET, Western Ontario Meniscal Evaluation Tool.

## DISCUSSION

The main finding in this study was that after a minimum follow‐up of 2 years, no statistically significant differences were found in clinical outcome's evolution between patients with and patients without preoperative pathological scores, thus rejecting our main hypothesis.

To our knowledge, the relationship between psychological factors and clinical outcome's evolution after FOCA transplantation of the knee has not been studied previously although it has been studied in other knee conditions. Domenech et al. [6], for example, performed a cross‐sectional study in 97 patients with chronic anterior knee pain (AKP). They observed that patients who had a high level of kinesiophobia, catastrophizing, anxiety, and depression had significantly more disability and pain than those with lower levels. Two years later, the same author published an observational study in 47 patients [5] with chronic AKP treated with either physical therapy or surgery and observed that a clinical improvement in pain and disability was associated with a decrease in pain catastrophizing and kinesiophobia. Unlike the findings in these mentioned studies, we did not find any relationship between our preoperative psychological scores and the evolution of postoperative clinical outcome after FOCA transplantation.

Regarding patients who undergo total knee arthroplasty (TKA), pain catastrophizing has been reported to be a significant factor in several post‐operative outcomes. According to Riddle et al. [33], who recruited 140 patients with moderate to high levels of pain and catastrophizing before a TKA procedure, pain catastrophizing was the only consistent psychological predictor of poor WOMAC outcome. In a two‐year follow‐up study of 55 patients who underwent TKA, Forsythe et al. [9] reported a greater incidence of pain in patients with abnormal preoperative PCS scores. Their observation also contrasts with our study where we observed that the incidence of preoperative pathological PCS scores had no influence on the evolution of clinical outcome after FOCA transplantation.

In relation to anxiety and depression scores, in a prospective cohort study of 186 patients Ali et al. [1] observed that patients who were undergoing a primary TKA and had preoperative anxiety or depression had a more than 6 times higher risk of post‐surgical dissatisfaction than patients with no anxiety or depression (*p* < 0.001). In another prospective study in 104 patients undergoing TKA, Hirschmann et al. [23] found that patients with lower preoperative psychological characteristics had lower WOMAC scores, KSS scores and VAS pain and satisfaction at 6 weeks, 4 months and 1 year after TKA. In our study, we did not detect any differences in clinical evolution after the FOCA procedure between those who had and those who did not have preoperative pathological scores on the HADS questionnaires.

Lastly, based on a study [8] that included 200 patients after TKA, Filardo et al. [8] found kinesiophobia was an independent factor influencing clinical outcomes according to the WOMAC questionnaire when administered at 12 months postoperatively. In contrast, in our study, we did not find any relationship between our preoperative TSK scores and the evolution of postoperative clinical outcomes after FOCA transplantation.

Although our results show that pathological scores on HADS, TSK and PCS questionnaires had no influence on clinical outcome's evolution after FOCA transplantation of the knee from of WOMET, Kujala, the IKDC score and the Tegner scale, psychological factors are now being considered in a number of surgical scenarios, and pre‐operative optimisation of psychological factors is increasingly performed [9].

Findings suggest that patients who undergo major knee surgery such as FOCA transplantation should be approached and optimised psychologically, nutritionally and physically in order to achieve the best possible results and in order to promote health enhance their resilience for their surgical recovery.

The main limitation of our study is that the sample size was small compared with the majority of studies discussed, since these are about more frequent pathologies. In relation to studies on FOCA, the sample size is comparable to previously published series on FOCA procedures. In addition, the cohort was relatively heterogeneous with respect to osteochondral allograft type. However, no statistically significant differences were noted for subgroup analysis.

## CONCLUSIONS

There were no significant differences between patients with pathological psychological scores and patients with non‐pathological psychological scores in terms of postoperative clinical outcome evolution, thus rejecting our main hypothesis.

## AUTHOR CONTRIBUTIONS

All authors contributed to the study conception and design. Material preparation, data collection and analysis were performed by Pablo Eduardo Gelber, Eduard Ramírez‐Bermejo and Anna Castella Pujol. The first draft of the manuscript was written by Anna Castella Pujol and all authors commented on previous versions of the manuscript. All authors read and approved the final manuscript.

## CONFLICT OF INTEREST STATEMENT

The authors declare no conflicts of interest.

## ETHICS STATEMENT

The study was approved by the clinical research ethics committee at our institution (IIBSP‐ALO‐2018‐21) and conducted in accordance with the ethical standards laid down in the 1964 Declaration of Helsinki and its later amendments. Informed consent was obtained from all individual participants included in the study.

## Supporting information

Supporting information.

## Data Availability

The data sets used or analysed during the current study are available from the corresponding author on reasonable request.
